# Assessing the impact of MSH3 and MSH6 polymorphisms on lung cancer risk in North Indian patients undergoing platinum chemotherapy through molecular dynamics simulation

**DOI:** 10.1038/s41598-024-67090-x

**Published:** 2024-07-13

**Authors:** Sidhartha Singh, Navneet Singh, Parth Sarthi Sen Gupta, Saroj Kumar Panda, Isha Dhamija, Deepak Nathiya, Sandeep Kumar, Siddharth Sharma

**Affiliations:** 1 Department of Biosciences and Bioengineering, D Y Patil International University , Akurdi, Pune, Maharashtra 411044 India; 2 Department of Pulmonary Medicine, Post Graduate Institute of Medical Education & Research (PGIMER), Chandigarh, India; 3 Department of Chemical Sciences, Indian Institute of Science Education and Research (IISER), Berhampur, India; 4 Department of Pharmacology and Toxicology, National Institute of Pharmaceutical Education and Research-Hyderabad (NIPER-H), Hyderabad, Telangana India; 5 Department of Pharmacy Practice, Nims Institute of Pharmacy, University Rajasthan, Jaipur, Rajasthan 303121 India; 6 Department of Pharmaceutics, Nims Institute of Pharmacy, Nims University Rajasthan, Jaipur, Rajasthan 303121 India; 7 Department of Biotechnology, Thapar Institute of Engineering & Technology, Patiala, Punjab 147004 India

**Keywords:** Lung cancer, Polymorphism, Overall survival, Chemotherapy, MSH3, MSH6, Cancer, Cell biology, Genetics, Molecular biology

## Abstract

The present study investigated the relationship between *MSH3* and *MSH6* genes in lung cancer patients. Genotyping of lung cancer patients and healthy controls was performed. Odds ratio values were calculated and survival analysis performed. Patients with mutant genotype (TT) for *MSH6* polymorphism have 1.5-fold risk for the development of lung cancer (*p* = 0.03). For non-smokers, the mutant-type genotype had a threefold increased risk of lung cancer (*p* = 0.01). Patients administered with docetaxel and carbo/cisplatin and carrying GT genotype for *MSH6* polymorphism, patients reported a decrease in median survival time (4.9 vs 9.13 months). *MSH3* and *MSH6* polymorphisms are involved in modulating the risk towards lung cancer. *MSH6* polymorphism is associated with high mortality rate for patients undergoing cisplatin and docetaxel chemotherapy.

## Introduction

Lung cancer is one of the most prevalent and leading causes of malignancy-related deaths worldwide, especially in developed countries^1^. The significant factors contributing to making lung cancer incurable are the failure of early detection and continuous exposure to carcinogens. Berz and coworkers have shown that if we can succeed in the early detection of cancer, then the probability of a successful treatment dramatically increases to 70% from 5%; also, Cassidy has shown that only 11% of people exposed to tobacco smoke will eventually develop this disease^2,3^. One of the ways which can lead to early detection of lung cancer and also understand why all individuals exposed to carcinogens are not developing lung cancer is to delve into the realm of genetic polymorphism. Epidemiological studies have demonstrated that individuals having alterations in a particular gene may have a high risk of developing a specific type of cancer and also why some individuals have less probability of developing cancer even though they are exposed to carcinogens^4^. This genetic susceptibility may occur due to inherited polymorphism in genes involved in carcinogen metabolism and DNA mismatch repair (MMR)^5,6^.

MMR is a DNA repair pathway responsible for recognizing and repairing the errors (insertion, deletion and misincorporation of bases) occurring during DNA replication and recombination. An increase of 50–10,000 folds in spontaneous mutability has been recorded if the MMR pathway is genetically inactive^7^. The role of the *MSH3* gene in the pathogenesis of cancer was first explained by Benachenhou and coworkers when they demonstrated that mutation in *hMSH3* may be involved in tumorigenesis^8^. *hMSH3* gene is located on chromosome 5q14.1, is composed of 1137 amino acids and has a molecular weight of 127 kDa. It heterodimerizes with MSH2 to form MutS β, which binds to DNA mismatches, thereby initiating DNA repair^9^. MSH6 protein acts as one of the critical components of the mismatch repair system encoded by the *MSH6* gene located on chromosome 2p16.3. It comprises 1360 amino acid residues and has a molecular mass of 152 kDa. The structure of MSH6 and MSH3 protein can be altered by any polymorphism present in their respective gene, which will render this protein non-functional due to which it will not be able to recognize DNA mismatch and thereby fails to rectify it, which will eventually lead to cancer^10^. NCBI’s SNP database currently has more than three hundred and fifty 2053 single nucleotide polymorphisms (SNPs), which lie under clinical significance in the *MSH3* and *MSH6* genes, respectively. Among these SNPs, rs26279 G > A polymorphism for *MSH3* and 557G > T polymorphism for *MSH6* is most frequently studied in various population and have been associated with carcinogenesis^11^. rs26279 (*Ala*^*1045*^*Thr*) is located on exon 23 and leads to G→A transition (G3133A), thus resulting in alanine (Ala) to threonine (Thr) amino acid change^12^. The changes in amino acid led to the development of mutant MSH3 protein, which cannot rectify DNA mismatches. Several studies have demonstrated that polymorphism in *MSH3* and *MSH6* genes are related to the development of various cancers, including breast, head and neck cancer and hepatocellular carcinoma^12–14^. A study by Xu and coworkers reported that rs26279 polymorphism could be used as a prognostic marker for NCSLC patients undergoing platinum-based chemotherapy. However, they could not find any association between *MSH6* polymorphism and the development of lung cancer^15^. So far as our knowledge is concerned, no study has been evaluated in Indian lung cancer patients to evaluate the role of the *MSH3* and *MSH6* polymorphism towards lung cancer susceptibility and as a prognostic marker. Hence, in this study, we have conducted a case–control study to investigate the association of *rs26279 G* > *A* and *rs3136228 G* > *T* polymorphism towards lung cancer. We have also evaluated the polymorphisms mentioned above and their role in clinic-pathological parameters, response rates and overall survival (OS) of patients undergoing platinum-based doublet chemotherapy.

## Material and method

### Study population and follow-up

In this investigation, 500 individuals diagnosed with lung cancer were enlisted from the Department of Pulmonary Medicine at the Postgraduate Institute of Medical Education and Research (PGIMER) in Chandigarh. The ethical review boards of both PGIMER and Thapar Institute of Engineering and Technology (TIET), Patiala, granted approval for this study, assigning it the approval number PGI/IEC/2014/305. Following the acquisition of written informed consent, approximately 4–5 ml of peripheral blood and additional epidemiological information were gathered from all participants. The selection of participants for this study was conducted impartially, with criteria including: (1) confirmation of NSCLC/SCLC, (2) diagnosis of stage III or IV lung cancer, (3) a performance status of 0–4 on the Eastern Cooperative Oncology Group performance status (ECOG) scale, and (4) written consent from the subjects. Demographic information such as age, gender, and smoking habits was documented for all participants.

The Healthy controls were selected on the basis of same age group and demography as of cases and without any morbidities.

### Chemotherapeutic regimen

The initial phase of chemotherapy involved the use of platinum-based drugs such as cisplatin and carboplatin, while the subsequent phase incorporated non-platinum-based medications like docetaxel, irinotecan, pemetrexed, and paclitaxel. This combined treatment was administered to all participants in the study. The drugs were intravenously infused every 3 weeks, with specific concentrations for each drug: 75, 500, 75, and 75 mg/m^2^ for docetaxel, pemetrexed, irinotecan, and paclitaxel, respectively, followed by a 3-h infusion of cisplatin at 70 mg/m^2^. Additionally, all patients received normal folate and vitamin B12 supplements. Prior to each chemotherapy cycle, a comprehensive blood count and metabolic profile were conducted. Patients underwent a maximum of six chemotherapy cycles, and after the fourth cycle, disease response was evaluated through computed tomographic scans, employing the Response Evaluation Criteria in Solid Tumors (RECIST) criteria. Adverse events (AEs) were documented and categorized according to the standard toxicity criteria (CTC) version 3.0

### Follow-up and response determination

All the recruited patients were telephonically followed up after two months until the end of the investigation or the patient’s death. Relatives of the patients and patients themselves provided the survival data. The survival was done till the last date of study or till the patient’s death, and survival time was calculated from the date of the patient’s enrolment till the last day of follow-up. Response Evaluation Criteria for Solid Tumors (RECIST) was used for the evaluation of tumour response, and based on that; patients were divided into four groups: patients showing complete response (CR), partial response (PR), stable disease (SD) and progression disease (PD). Further, these categories are grouped into the “responders” –patients showing CR and PR and “non-responders”- patients showing SD and PD.

### Genotyping of *MSH3* and *MSH6* variants

**Figure 1:**
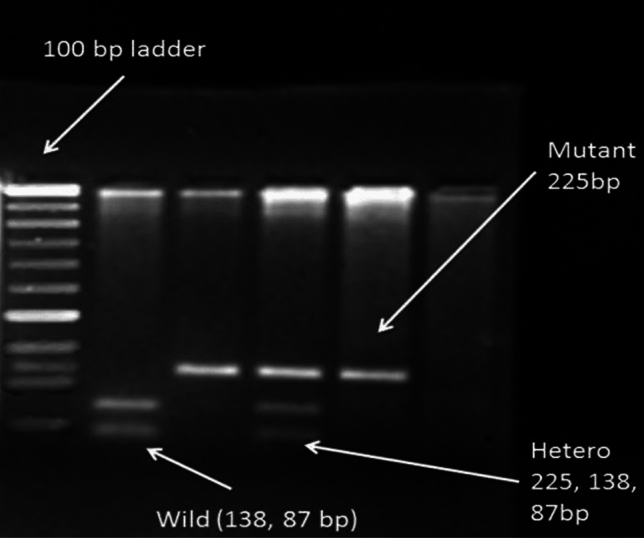
Restriction digestion pattern for MHS3 (rs26279) variants; wild type (AA) 138, 87 bp; mutant type (GG): 225 bp; heterozygous type (AG): 225, 137, 87 bp. Lane 1: Marker; Lane 2: AA genotype, Lane 3: GG genotype; Lane 4: GA genotype.

**Figure 2:**
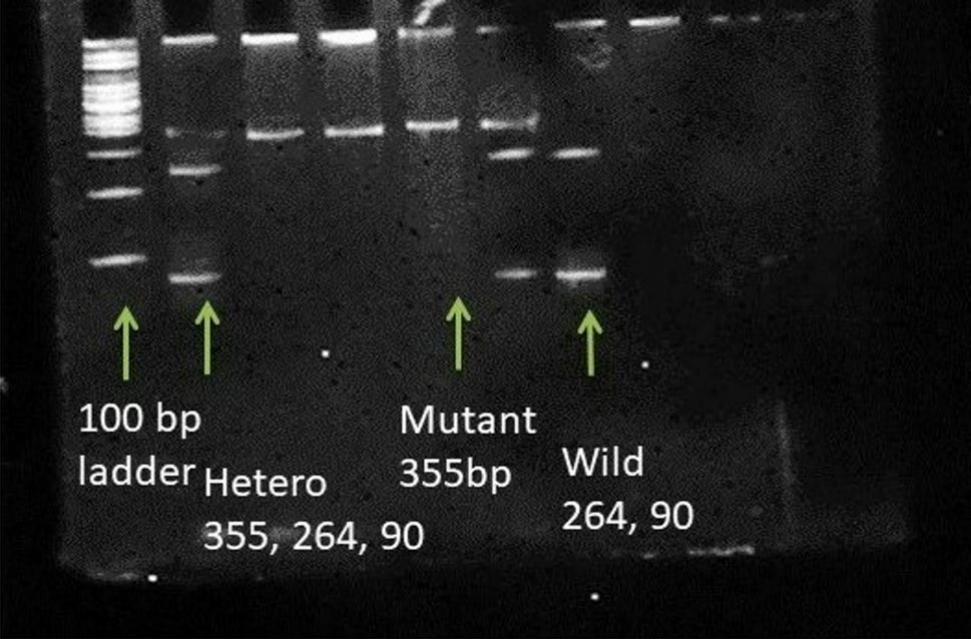
Restriction digestion pattern for *MSH6* (557G > T, rs3136228) polymorphism. Band size: *GG*: 264, 90 bp; *GT*: 355, 264, 90 bp; *TT*: 355 bp.

Genomic DNA was isolated from 4 ml of blood using a phenol–chloroform extraction procedure described by singh and colleagues^16^. The genotype of *MSH3* and *MSH6* was determined by polymerase chain reaction–restriction fragment length polymorphism (PCR–RFLP) assay. The primers used to amplify *MSH3* variants were FP: 5′-TCTAACAGGCAAGTAGGAAC-3′ RP: 5′-TAGCCA CATTTAATCCATAAC-3′ and for *MSH6* variants were: FP: 5′-GGCTCAGATAACGGACTG TGG- 3′, RP: 5′-ACCCGAAAGGCCTCGGAAAG-3′. The PCR master mix (20 μl) is composed of 1X PCR buffer, 100 μg/ml bovine serum albumin (BSA), 0.5 μM of forward and reverse primer, 1.5 mM MgCl_2_, 200 μM dNTPs, and 1U Taq polymerase and 100 ng template DNA. The PCR was run under the following conditions: denaturation step—5 min at 95 °C and 30 s at 94 °C, annealing step—45 s at 63 °C (*MSH3*) and 65 °C (*MSH6*), extension step—29 cycles for 30 s each at 72 °C and the final extension step—5 min at 72 °C. The length of the PCR product for *MSH3* variant 225 bp was further confirmed by agarose gel electrophoresis using a gel concentration of 1.5%. The polymorphic variants for *MSH3* and *MSH6* genes were analyzed after digesting the PCR product with HhaI (Takara) and Msp I at 37 °C overnight and running the digested product in 2.5% agarose gel (as shown in Fig. 1). For *MSH3,* the mutant allele produced a single band of 225 bp, the wild allele produced two bands of 138 & 87 bp, and the heterozygous allele produced three bands of 225, 138 &87 bp. For *MSH6,* the wild allele produced a single band of 355 bp, the mutant allele produced two bands of 264 and 90 bp, and the heterozygous allele produced three bands of 355, 264 and 90 bp (as shown in Fig. 2). Two individuals checked the banding pattern to remove any bias; moreover, 20% of the randomly selected samples were repeated to check the reproducibility, which was found to be 100%.

### Statistical analysis

This study concentrated on the population residing in north India, collecting information on sex, age, and smoking habits. To assess the alignment of cases and controls, the study employed the Hardy–Weinberg equilibrium and the goodness-of-fit Chi-square test. The odds ratio (OR) with a 95% confidence interval (CI) and *p* < 0.05 was calculated using the unconditional logistic regression method, incorporating age, sex, and smoking status as confounding factors. All statistical analyses were conducted using MedCalc statistical software version 14.8.1 (MedCalc Software, Ostend, Belgium). Various genetic models (Co-dominant, Dominant, and Recessive) were utilized in this study. In genetic association studies, the ability to detect disease-associated SNPs depends on several factors, including the genetic models tested. Maximum statistical power is achieved when the mode of inheritance of the disease-associated SNP aligns with the genetic model used. Kaplan–Meier method determined the median OS, with a log-rank *p*-value less than 0.05 considered as significant. The multivariate Cox regression method estimated OS, accounting for confounding factors such as age, sex, tumor stage, histology, Eastern Cooperative Oncology Group (ECOG), and smoking. Additionally, the hazard ratio (HR) was calculated using both Kaplan–Meier and Cox methods to assess the relationship between OS and MSH3 polymorphism based on the chemotherapeutic regimen. Tumor response was evaluated using the Response Evaluation Criteria for Solid Tumors (RECIST).

### MD simulations

Here apo-msh3 and the mutant *A1045T*, were chosen for additional molecular dynamics research. In this case, GROMACS 2022 was used to conduct 100-ns long MD simulations. As in our earlier work, we adapted all of the standard procedures for MD simulations^17–22^. The AMBERff99SB^22^ force field was used to achieve the optimal folding of the protein. Through the addition of an appropriate quantity of Na+ and Cl−, the protein was both solvated and electrically neutralized. In addition, the energy consumption of the entire system was brought down to a minimum by employing the steepest descent approach for 50,000 steps. Again, all of the systems were brought to a state of equilibrium by employing the canonical (NVT) approach and the isothermal-isobaric ensemble (NPT) method for a period of 5 ns each. After that, the final simulations for both systems were ran for a duration of 100 ns.

After the simulations were finished, the trajectories were extracted, and all of the post-molecular dynamics investigations were carried out. This was done in order to assess the stability of those protein systems as well as any changes in their conformation. In this section, we have computed a variety of parameters, such as the root-mean-square-deviation (RMSD), the root-mean-square-fluctuation (RMSF), the radius of gyration (Rg), the solvent accessible surface area (SASA), the hydrogen bonds (H-bond), and the principal component analysis (PCA).

## Results

### Patient characteristics and clinical predictors

The study included 500 lung cancer patients and 500 healthy controls. Key characteristics such as age, sex, smoking habits, pack years, histology, TNM staging, and other clinical data are detailed in Table 1. The average age of the control group was 61.6 ± 11.4 years, while the cancer patients averaged 60.5 ± 9.86 years. Among the 500 lung cancer patients, 41% had squamous-cell carcinoma (SQCC), 40.6% had adenocarcinoma (ADCC), and 16.8% had small-cell lung carcinoma (SCLC). The TNM stage distribution among the patients was: stage I: 0.2%, stage II: 3.8%, stage III: 38.6%, and stage IV: 51.4%. Tumor sizes T3 and T4 were significantly more common than T1 and T2 (387 vs 67). For lymph node involvement, 13% of patients were N0, while 6.8%, 42%, and 32.6% were N1, N2, and N3, respectively. Regarding metastasis, 42% of patients had no metastasis (M0), and 52.6% had distant metastasis (M1). Performance status was assessed using KPS and ECOG scores: about 18% had a KPS below 60, while 54.4% and 27.5% had KPS scores of 70–80 and 90–100, respectively. ECOG scores showed that 44.9% had values between 0–1, 39.49% had a score of 2, and 15.54% had scores between 3 and 4. As shown in Table 1, 354 of the cancer patients received platinum-based doublet chemotherapy. Of these, 29.66% received pemetrexed with cisplatin/carboplatin, 12.71% received irinotecan with cisplatin/carboplatin, 10.45% received docetaxel with cisplatin/carboplatin, 23.16% received paclitaxel with cisplatin/carboplatin, and 8.19% received gemcitabine with cisplatin/carboplatin.

**Table 1:** Demographic characteristics among cases and controls.

Variable	Total (N)	Cases, n (%)	Controls, n (%)	^a^*p-*value
Age (years)	500			
Mean ± SD		60.5 ± 9.86	61.6 ± 11.4	0.103
Range		30–86	31–83	
Gender	500			
Male		401 (80.2)	455 (91.0)	
Female		99 (19.8)	45 (9.0)	**< 0.0001**
Smoking status	500			
Smokers		398 (79.6)	449 (89.8)	
Non-smokers		102 (20.4)	51 (10.2)	**< 0.0001**
Pack years	500			
Mean ± SD		20.82 ± 21.03	13.84 ± 11.27	**< 0.0001**
Histological types	500			
SQCC		205 (41.0)		
ADCC		203 (40.6)		
SCLC		84 (16.8)		
Others		6 (1.2)		
Unknown		2 (0.4)		
Overall survival	475			
Dead		356 (74.9)		
Alive		119 (25.0)		
Staging	475			
I		6 (1.2)		
II		19 (4.0)		
III		193 (40.6)		
IV		257 (54.1)		
Unclassified		25 (5.2)		
Tumor size	475			
Tx		19 (4.0)		
T1		26 (5.4)		
T2		41 (8.6)		
T3		85 (17.8)		
T4		302 (63.5)		
Unknown		27 (5.6)		
Lymph node	475			
Nx		3 (0.6)		
N0		65 (13.6)		
N1		34 (7.15)		
N2		210 (44.2)		
N3		163 (34.3)		
N4		0 (0)		
Unknown		25 (5.2)		
Metastasis	475			
Mx		1 (0.2)		
M0		210 (44.2)		
M1		263 (55.3)		
Unknown		26 (5.4)		
Performance status	476			
KPS (below 60)		86(18.1)		
KPS (70–80)		259(54.4)		< 0.0001
KPS (90–100)		131(27.5)		< 0.0001
ECOG (0–1)	476	214(44.9)		
ECOG (2)		188(39.49)		< 0.0001
ECOG (3–4)		74(15.54)		< 0.0001
Chemotherapy regimen	354			
Pemetrexed cis/carboplatin		105(29.66)		
Irinotecan cis/carboplatin		45(12.71)		
Docetaxel cis/carboplatin		37(10.45)		
Paclitaxel cis/carboplatin		82(23.16)		
Gemcitabine cis/carboplatin		29(8.19)		
Ceretinib		1(0.28)		
Others		48(13.55)		

SD, standard deviation; n, total number of lung cancer cases or control subjects.

^a^*p*-values were derived from Pearson Chi-square test except age and pack-years; Student t-test was used for age and pack-years.

All *p*-values are two-sided. *p* < 0.05 was considered statistically significant.

### Association of the MSH3 Ala^1045^Thr and MSH6 557 G > T polymorphism with risk of lung cancer according to tumour histology

Table 2 shows the distribution of the MSH3 Ala1045Thr polymorphism in control subjects and lung cancer cases. In the control group, 71% were homozygous for the wild-type genotype (AA), 3.6% were homozygous variant carriers (GG), and 25.4% had the heterozygous genotype (GA). In contrast, among lung cancer cases, 73.8% were homozygous for the wild-type genotype (AA), 23.4% had the heterozygous genotype (GA), and 2.8% were homozygous for the variant alleles (GG). There was a significant difference in the distribution of genotypic frequencies between cases and controls for the MSH3 polymorphism (χ^2^ = 1.81; df = 2; *p* = 0.03). For the MSH6 557 G > T polymorphism, the genotype frequencies in controls were 81.4% TT, 17% GT, and 1.6% GG, while in cases, they were 86.4% TT and 13.6% GT, with no GG homozygous variants found in the cases. There was no significant difference in the distribution of genotypic frequencies between cases and controls for the MSH6 557 G > T polymorphism (χ^2^ = 10.63; df = 2; *p* = 0.10). Both MSH3 and MSH6 polymorphic variants showed no deviation from Hardy–Weinberg equilibrium (HWE). For MSH3, the HWE values were {Cases: χ^2^ = 1.58; df = 1; *p* = 0.20; Controls: χ^2^ = 2.38; df = 1; *p* = 0.12}, and for MSH6, they were {Cases: χ^2^ = 2.66; df = 1; *p* = 0.10; Controls: χ^2^ = 2.03; df = 1; *p* = 0.15}. The minor allele frequency (MAF) for MSH3 was 0.145 in cases and 0.163 in controls, while for MSH6, it was 0.068 in cases and 0.101 in controls.

**Table 2:** Genotypic and allelic distribution of the *MSH3* and *MSH6* genetic variant and its association with risk of lung cancer overall and according to tumor histology.

Genotype rs26279 G > A (MSH3)	Controls (500) N (%)	Cases (500) N (%)	AOR (95% CI)^a^	*p*^b^	Genotype rs3136228 557 G > T (MSH6)	Controls (500) N (%)	Cases (500) N (%)	AOR (95% CI)^a^	*p*^b^
Co-dominant model					Co-dominant model				
OVERALL									
AA	355 (71.0)	369 (73.8)	1.00 (Reference)		TT	407 (81.4)	432 (86.4)	1.00 (Reference)	
GA	127 (25.4)	117 (23.4)	0.90 (0.67–1.21)	0.52	GT	85 (17)	68 (13.6)	0.75 (0.53–1.07)	0.12
GG	18 (3.6)	14 (2.8)	0.75 (0.36–1.56)	0.45	GG	8 (1.6)	0 (0)	0	0.99
G(Allele)	837	855			GG (Allele)	899	932		
A(Allele)	163	145			TT (Allele)	101	68		
MAF	0.163	0.145			MAF	0.101	0.068		
χ^2^ = 1.81; df = 2				0.03	χ^2^ = 10.63; df = 2				0.10
Dominant model					Dominant model				
AA	355 (71.0)	369 (73.8)	1.00 (Reference)		TT	407 (81.4)	432 (86.4)	1.00 (Reference)	
GA + GG	145 (29.0)	131 (26.2)	0.89 (0.67–1.18)	0.42	GT + GG	93 (18.6)	68 (13.6)	0.69 (0.49–0.98)	**0.03**
					Recessive model				
					GT + GG	93 (18.6)	68 (13.6)	1.00 (Reference)	
					TT	407 (81.4)	432(86.4)	1.43 (1.01–2.03)	**0.03**

Genotype rs26279 G > A (MSH3)	Controls (500) N (%)	Cases (203) N (%)	AOR (95% CI)^a^	*p*^b^	Genotype rs3136228 557 G > T (MSH6)	Controls (500) N (%)	Cases (203) N (%)	AOR (95% CI)^a^	*p*^b^
Co-dominant model					Co-dominant model				
ADCC									
AA	355 (71.0)	152 (74.9)	1.00 (Reference)		TT	407 (81.4)	177 (87.2)	1.00 (Reference)	
GA	127 (25.4)	48 (23.7)	0.99 (0.66–1.49)	0.97	GT	85 (17)	26 (12.8)	0.60 (0.36–1.02)	**0.06**
GG	18 (3.6)	3 (1.4)	0.40 (0.10–1.49)	0.17	GG	8 (1.6)	0 (0)	0	0.99
Dominant model					Dominant model				
AA	355 (71.0)	152 (74.8)	1.00 (Reference)		TT	407 (81.4)	177 (87.2)	1.00 (Reference)	
GA + GG	145 (29.0)	51 (25.2)	0.92 (0.61–1.37)	0.69	GT + GG	93 (18.6)	26 (12.8)	0.56 (0.34–0.95)	**0.03**
					Recessive model				
					GT + GG	93 (18.6)	26 (12.8)	1.00 (Reference)	
					TT	407 (81.4)	177 (87.2)	1.75 (1.05–2.93)	**0.03**

Genotype rs26279 G > A (MSH3)	Controls (500) N (%)	Cases (205) N (%)	AOR (95% CI)^a^	*p*^b^	Genotype rs3136228 557 G > T (MSH6)	Controls (500) N (%)	Cases (205) N (%)	AOR (95% CI)^a^	*p*^b^
Co-dominant model					Co-dominant model				
SQCC									
AA	355 (71.0)	146 (71.2)	1.00 (Reference)		TT	407 (81.4)	172 (83.9)	1.00 (Reference)	
GA	127 (25.4)	50 (24.3)	0.94 (0.64–1.37)	0.75	GT	85 (17)	33 (16.1)	1.01 (0.64–1.58)	0.96
GG	18 (3.6)	9 (4.3)	1.04 (0.45–2.42)	0.91	GG	8 (1.6)	0 (0)	0	0.99
Dominant model					Dominant model				
AA	355 (71.0)	146 (71.3)	1.00 (Reference)		TT	407 (81.4)	172 (83.9)	1.00 (Reference)	
GA + GG	145 (29.0)	59 (28.7)	0.95 (0.66–1.37)	0.80	GT + GG	93 (18.6)	33 (16.1)	0.91 (0.58–1.42)	0.69
					Recessive model				
					GT + GG	93 (18.6)	33 (16.1)	1.00 (Reference)	
					TT	407 (81.4)	172 (83.9)	1.09 (0.70–1.70)	0.69

Genotype rs26279 G > A (MSH3)	Controls (500) N (%)	Cases (84) N (%)	AOR (95% CI)^a^	*p*^b^	Genotype rs3136228 557 G > T (MSH6)	Controls (500) N (%)	Cases (84) N (%)	AOR (95% CI)^a^	*p*^b^
Co-dominant model					Co-dominant model				
SCLC									
AA	355 (71.0)	65 (77.4)	1.00 (Reference)		TT	407 (81.4)	75 (89.3)	1.00 (Reference)	
GA	127 (25.4)	18 (21.4)	0.80 (0.45–1.42)	0.44	GT	85 (17)	9(10.7)	0.63 (0.30–1.33)	0.22
GG	18 (3.6)	1 (1.2)	0.25 (0.03–2.02)	0.19	GG	8 (1.6)	0	0	0.99
Dominant model					Dominant model				
AA	355 (71.0)	65 (77.4)	1.00 (Reference)		TT	407 (81.4)	75 (89.3)	1.00 (Reference)	
GA + GG	145 (29.0)	19 (22.6)	0.72 (0.41–1.26)	0.25	GT + GG	93 (18.6)	9(10.7)	0.57 (0.27–1.21)	0.14
					Recessive model				
					GT + GG	93 (18.6)	9(10.7)	1.00 (Reference)	
					TT	407 (81.4)	75 (89.3)	1.73 (0.83–3.62)	0.14

^a^Adjusted Odds ratios, 95% confidence intervals and their corresponding *p*-values were calculated by logistic regression analysis after adjusting for age, gender.

^b^Two-sided χ^2^ test for either genotype distribution or allelic frequencies between the cases and controls. The number in bold indicates the significant values in the table.

To evaluate the association of MSH3 and MSH6 polymorphic variants with lung cancer, three genetic models were applied. Regression analysis was used to calculate the adjusted odds ratio (AOR) and 95% confidence interval (CI). For the co-dominant model of the MSH3 Ala1045Thr polymorphism, no significant association was found with lung cancer susceptibility (AOR 0.90; 95% CI 0.67–1.21; *p* = 0.52). The dominant model also showed no significant association between the MSH3 Ala1045Thr variant and lung cancer. Furthermore, no association was observed between the MSH3 variant and different histological subtypes of lung cancer.

For the MSH6 557G > T polymorphism, the co-dominant model showed no significant association with lung cancer susceptibility (AOR 0.75; 95% CI 0.53–1.07; *p* = 0.12). However, the dominant model predicted a decreased risk of developing lung cancer in the combined genotype (AOR 0.69; 95% CI 0.49–0.98; *p* = 0.03). Conversely, the recessive model predicted a 1.5-fold increased risk of lung cancer in individuals with the mutant genotype (AOR 1.43; 95% CI 1.01–2.03; *p* = 0.03). When lung cancer subjects were segregated based on histological subtypes, the co-dominant and dominant models for the MSH6 557G > T polymorphism predicted a decreased risk of developing adenocarcinoma in subjects with the heterozygous genotype (GT) (AOR 0.6; 95% CI 0.36–1.02; *p* = 0.06) and the combined (GT + GG) genotype (AOR 0.56; 95% CI 0.34–0.95; *p* = 0.03). No association was found between the MSH6 polymorphism and patients with SCLC or SQCC.

### Association of the ***MSH3 Ala***^***1045***^***Thr*** and ***MSH6*** 557 G > T polymorphism with smoking status

In our study, the number of smokers in the case and control groups were 398 and 449, respectively. These smokers were further divided into two subgroups: heavy smokers (pack years > 20) and light smokers (pack years ≤ 20) as shown in Table 3. For the MSH3 Ala1045Thr polymorphism, no association was found between smoking and the polymorphism, nor was there a significant difference between heavy and light smokers when stratified by smoking index. In contrast, for the MSH6 557 G > T polymorphism, a significant finding was observed when applying the co-dominant model. Non-smokers carrying the heterozygous genotype (GT) had a decreased risk of developing lung cancer (AOR 0.31, 95% CI 0.12–0.78, *p* = 0.01). However, in the recessive model, subjects with the TT genotype had a three-fold increased risk of developing lung cancer (AOR 3.22; 95% CI 1.26–8.18; *p* = 0.01) as shown in Table 3. No association was found between MSH3 and MSH6 polymorphisms and the propensity for lung cancer susceptibility among light and heavy smokers.

**Table 3:** Relationship of different *MSH3* and *MSH6* genotypes with the smoking status of cases and controls.

Genotype rs26279 G > A (MSH3)	Controls (449) N (%)	Cases (398) N (%)	AOR (95% CI)^a^	*p*^b^	Genotype rs3136228 557 G > T (MSH6)	Controls (449) N (%)	Cases (398) N (%)	AOR (95% CI)^a^	*p*^b^
Co-dominant model					Co-dominant model				
SMOKERS									
AA	313 (69.7)	292 (73.4)	1.00 (Reference)		TT	371 (82.7)	341 (85.7)	1.00 (Reference)	
GA	118 (26.3)	95 (23.9)	0.87 (0.63–1.19)	0.40	GT	70 (15.6)	57 (14.3)	0.91 (0.62–1.34)	0.64
GG	18 (4.0)	11 (2.7)	0.58 (0.26–1.30)	0.19	GG	8 (1.7)	0 (0)	0	0.99
Dominant model					Dominant model				
AA	313 (69.7)	292 (73.4)	1.00 (Reference)		TT	371 (82.6)	341 (85.7)	1.00 (Reference)	
GA + GG	136 (30.3)	106 (26.6)	0.83 (0.61–1.13)	0.24	GT + GG	78 (17.4)	57 (14.3)	0.82 (0.56–1.19)	0.30
					Recessive model				
					GT + GG	78 (17.3)	57 (14.3)	1.00 (Reference)	
					TT	371 (82.7)	341 (85.7)	1.21 (0.83–1.77)	0.30

Genotype rs26279 G > A (MSH3)	Controls (51) N (%)	Cases (102) N (%)	AOR (95% CI)^a^	*p*^b^	Genotype rs3136228 557 G > T (MSH6)	Controls (51) N (%)	Cases (102) N (%)	AOR (95% CI)^a^	*p*^b^
Co-dominant model					Co-dominant model				
NON SMOKERS									
AA	42 (82.4)	77 (75.5)	1.00 (Reference)		TT	36 (70.6)	91 (89.3)	1.00 (Reference)	
GA	9 (17.6)	22 (21.6)	1.47 (0.57–3.75)	0.41	GT	15 (29.4)	11 (10.7)	0.31 (0.12–0.78)	**0.01**
GG	0 (0)	3 (2.9)	2.48E + 07	0.99	GG	0	0	0	0.99
Dominant model					Dominant model				
AA	42 (82.4)	77 (75.5)	1.00 (Reference)		TT	36 (70.6)	91 (89.3)	1.00 (Reference)	
GA + GG	9 (17.6)	25 (24.5)	1.65 (0.65–4.15)	0.28	GT + GG	15 (29.4)	11 (10.7)	0.31 (0.12–0.78)	**0.01**
					Recessive model				
					GT + GG	15 (29.4)	11 (10.7)	1.00 (Reference)	
					TT	36 (70.6)	91 (89.3)	3.22 (1.26–8.18)	**0.01**

Genotype rs26279 G > A (MSH3)	Controls (323) N (%)	Cases (155) N (%)	AOR (95% CI)^a^	*p*^b^	Genotype rs3136228 557 G > T (MSH6)	Controls (323) N (%)	Cases (155) N (%)	AOR (95% CI)^a^	*p*^b^
Co-dominant model					Co-dominant model				
LIGHT SMOKERS									
AA	222 (68.7)	117 (75.6)	1.00 (Reference)		TT	269 (83.3)	131 (84.5)	1.00 (Reference)	
GA	88 (27.3)	34 (21.9)	0.75 (0.47–1.20)	0.23	GT	49 (15.2)	24 (15.5)	1.06 (0.61–1.82)	0.82
GG	13 (4.0)	4 (2.5)	0.39 (0.10–1.44)	0.16	GG	5 (1.5)	0	0	0.99
Dominant model					Dominant model				
AA	222 (68.7)	117 (75.5)	1.00 (Reference)		TT	269 (83.3)	131 (84.5)	1.00 (Reference)	
GA + GG	101 (31.3)	38 (24.5)	0.70 (0.45–1.10)	0.13	GT + GG	54 (16.7)	24 (15.5)	0.96 (0.56–1.64)	0.89
					Recessive model				
					GT + GG	54 (16.7)	24 (15.5)	1.00 (Reference)	
					TT	269 (83.3)	131 (84.5)	1.03 (0.60–1.77)	0.89

Genotype rs26279 G > A (MSH3)	Controls (126) N (%)	Cases (243) N (%)	AOR (95% CI)^a^	*p*^b^	Genotype rs3136228 557 G > T (MSH6)	Controls (126) N (%)	Cases (243) N (%)	AOR (95% CI)^a^	*p*^b^
Co-dominant model					Co-dominant model				
HEAVY SMOKERS									
AA	91 (72.2)	175 (72.0)	1.00 (Reference)		TT	102 (80.9)	210 (86.4)	1.00 (Reference)	
GA	30 (23.9)	61 (25.2)	1.05 (0.63–1.75)	0.83	GT	21 (16.7)	33 (13.6)	0.77 (0.42–1.41)	0.41
GG	5 (3.9)	7 (2.8)	0.75 (0.23–2.45)	0.64	GG	3 (2.4)	0	0	0.99
Dominant model					Dominant model				
AA	91 (72.3)	175 (72.1)	1.00 (Reference)		TT	102 (80.9)	210 (86.4)	1.00 (Reference)	
GA + GG	35 (27.7)	68 (27.9)	1.01 (0.62–1.63)	0.96	GT + GG	24 (19.1)	33 (13.6)	0.68 (0.38–1.22)	0.19
					Recessive model				
					GT + GG	24 (19.1)	33 (13.6)	1.00 (Reference)	
					TT	102 (80.9)	210 (86.4)	1.46 (0.81–2.61)	0.19

^a^Adjusted Odds ratios, 95% confidence intervals and their corresponding *p*-values were calculated by logistic regression analysis after adjusting for age, gender.

^b^Two-sided χ2 test for either genotype distribution or allelic frequencies between the cases and controls. The number in bold indicates the significant values in the table.

### Association of ***MSH3 Ala***^***1045***^***Thr*** and ***MSH6*** 557 G > T polymorphism with gender

A univariate analysis was conducted to estimate the association between gender and the MSH3 Ala1045Thr polymorphism in the occurrence of lung cancer, as shown in Table 4. When the co-dominant model was applied, the data indicated that female lung cancer patients who were heterozygous carriers (GA) had a 2.35-fold increased risk (95% CI 0.85–6.52; *p* = 0.04) of developing lung cancer. This trend was also evident in the dominant model, where female subjects exhibited a 2.4-fold increased risk (OR 2.39, 95% CI 0.90–6.23; *p* = 0.03) of developing lung cancer (Table 4). For the MSH6 557G > T polymorphism, no association was found between gender and the risk of developing lung cancer.

**Table 4:** Relationship of different *MSH3* and *MSH6* polymorphisms on the basis of gender.

Genotype rs26279 G > A (MSH3)	Controls (455) N (%)	Cases (401) N (%)	AOR (95% CI)^a^	*p*^b^	Genotype rs3136228 557 G > T (MSH6)	Controls (455) N (%)	Cases (401) N (%)	AOR (95% CI)^a^	*p*^b^
Co-dominant model					Co-dominant model				
MALE									
AA	318 (69.9)	298 (74.4)	1.00 (Reference)		TT	370 (81.4)	342 (85.3)	1.00 (Reference)	
GA	120 (26.4)	93 (23.2)	0.84 (0.61–1.16)	0.31	GT	77 (16.9)	59 (14.7)	0.78 (0.53–1.14)	0.20
GG	17 (3.7)	10 (2.4)	0.64 (0.28–1.43)	0.27	GG	8 (1.7)	0 (0)	0	0.99
Dominant model					Dominant model				
AA	318 (69.8)	298 (74.4)	1.00 (Reference)		TT	370 (81.4)	342 (85.3)	1.00 (Reference)	
GA + GG	137 (30.2)	103 (25.6)	0.82 (0.61–1.11)	0.21	GT + GG	85 (18.6)	59 (14.7)	0.71 (0.49–1.03)	0.07
					Recessive model				
					GT + GG	85 (18.6)	59 (14.7)	1.00 (Reference)	
					TT	370 (81.4)	342 (85.3)	1.40 (0.96–2.02)	0.07

Genotype rs26279 G > A (MSH3)	Controls (45) N(%)	Cases (99) N(%)	AOR (95% CI)^a^	*p*^b^	Genotype rs3136228 557 G > T (MSH6)	Controls (45) N(%)	Cases (99) N(%)	AOR (95% CI)^a^	*p*^b^
Co-dominant model					Co-dominant model				
FEMALE									
AA	37 (82.3)	71 (71.7)	1.00 (Reference)		TT	37 (82.2)	90 (90.9)	1.00 (Reference)	
GA	**7 (15.5)**	**24 (24.2)**	**2.35 (0.85–6.52)**	**0.04**	GT	8 (17.8)	9 (9.1)	0.59 (0.17–1.51)	0.22
GG	1 (2.2)	4 (4.1)	2.11 (0.19–22.0)	0.53	GG	0 (0)	0 (0)	0	0.99
Dominant model					Dominant model				
AA	37 (82.3)	71 (71.7)	1.00 (Reference)		TT	37 (82.2)	90 (90.9)	1.00 (Reference)	
GA + GG	**8 (17.7)**	**28 (28.3)**	**2.39 (0.90–6.23)**	**0.03**	GT + GG	8 (17.8)	9 (9.1)	0.59 (0.17–1.51)	0.22
					Recessive model				
					GT + GG	8 (17.8)	9 (9.1)	1.00 (Reference)	
					TT	37 (82.2)	90 (90.9)	1.95 (0.65–5.79)	0.22

^a^Adjusted Odds ratios, 95% confidence intervals and their corresponding *p*-values were calculated by logistic regression analysis after adjusting for age, gender.

^b^Two-sided χ2 test for either genotype distribution or allelic frequencies between the cases and controls. The number in bold indicates the significant values in the table.

### Association of the ***MSH3 Ala***^***1045***^***Thr*** and ***MSH6 557G*** > ***T*** polymorphism & clinic-pathological parameter

The impact of the MSH3 Ala1045Thr and MSH6 557G > T variants on various clinicopathological parameters, such as stage, tumor extension, lymph node invasion, and metastasis, was assessed (Supplementary Table 1). Patients were classified by tumor stage (III and IV), tumor extension (T3 and T4), lymph node invasion (Nx + N0 + N1 and N2 + N3 + N4), and metastatic status (M0 and M1). No association was found between the MSH3 Ala1045Thr and MSH6 557G > T polymorphisms and these clinicopathological features, including tumor stage, size, lymph node invasion, and metastatic status.

### Association of ***MSH3 Ala***^***1045***^***Thr*** and ***MSH6*** 557 G > T polymorphism and chemotherapy response

Univariate logistic regression analysis was used to estimate the association between the MSH3 Ala1045Thr and MSH6 557 G > T polymorphisms and the response rate to chemotherapy (Supplementary Table 2). Patients were classified into two groups based on their response to chemotherapy: good responders (complete or partial remission, CR + PR) and inadequate responders (progressive or stable disease, PD + SD). No significant difference was observed in the chemotherapy response across all groups (*p* = 0.30). Therefore, the MSH3 Ala1045Thr and MSH6 557G > T polymorphic variants were not found to be predictors of the chemotherapy response rate.

### Survival analysis of ***MSH3 Ala***^***1045***^***Thr*** and ***MSH6*** 557 G > T genotype

Survival analysis and the association with MSH3 Ala1045Thr and MSH6 557G > T polymorphisms in 475 lung cancer cases are presented in Supplementary Table 3. Univariate analysis was conducted using the Kaplan–Meier method, and multivariate analysis was performed using Cox regression analysis, adjusting for age, sex, smoking status, stage, and ECOG, to evaluate any association between these polymorphisms and the survival of lung cancer patients. The median survival time for lung cancer patients with the MSH3 Ala1045Thr homozygous variant (GG) was higher than for those with the wild-type genotype (AA) (MST = 16.7 vs 8.7, 95% CI 0.32–1.05, Log-rank *p* = 0.14). However, in the multivariate analysis, no significant association between overall survival (OS) and the MSH3 Ala1045Thr or MSH6 557G > T polymorphisms was found after adjusting for confounding factors.

Additionally, the prognosis of lung cancer patients was evaluated based on the genotypes of MSH3 and MSH6, stratified by histological subtypes. No significant association was observed between survival rates and histological subtypes (ADCC, SQCC, and SCLC) (Supplementary Table 3).

### Association of ***MSH3 Ala***^***1045***^***Thr*** and ***MSH6*** 557 G > T polymorphism with chemotherapy regimens and OS

All lung cancer patients selected for this study were administered platinum-based doublet chemotherapy (carboplatin/cisplatin) as first-line therapy, along with other chemotherapeutic agents used in second-line treatment, such as paclitaxel, pemetrexed, irinotecan, and docetaxel. We aimed to evaluate the relationship between the MSH3 Ala1045Thr and MSH6 557G > T polymorphisms and overall survival, to determine if there was any association between overall survival, different chemotherapeutic regimens, and these polymorphisms. The results regarding the impact of MSH3 and MSH6 polymorphisms on overall survival according to chemotherapy regimen are shown in Supplementary Table 4.

No significant association was found between survival and the use of carboplatin/cisplatin with irinotecan, paclitaxel, and pemetrexed for the MSH3 and MSH6 variants. However, lung cancer patients with a single copy of the variant allele (GT) for the MSH6 557G > T polymorphism who received docetaxel along with first-line chemotherapy had a poorer median survival time compared to those with the TT genotype receiving the same regimen (MST = 4.9 vs 9.13, Log-rank *p* = 0.02). The Cox regression model analysis for the MSH6 polymorphism showed a two-fold increase in the hazard ratio and a corresponding poor outcome for these lung cancer patients (HR = 2.28; MST = 4.9; *p* = 0.03) (Supplementary Fig. 1).

### Molecular dynamics

**Figure 3:**
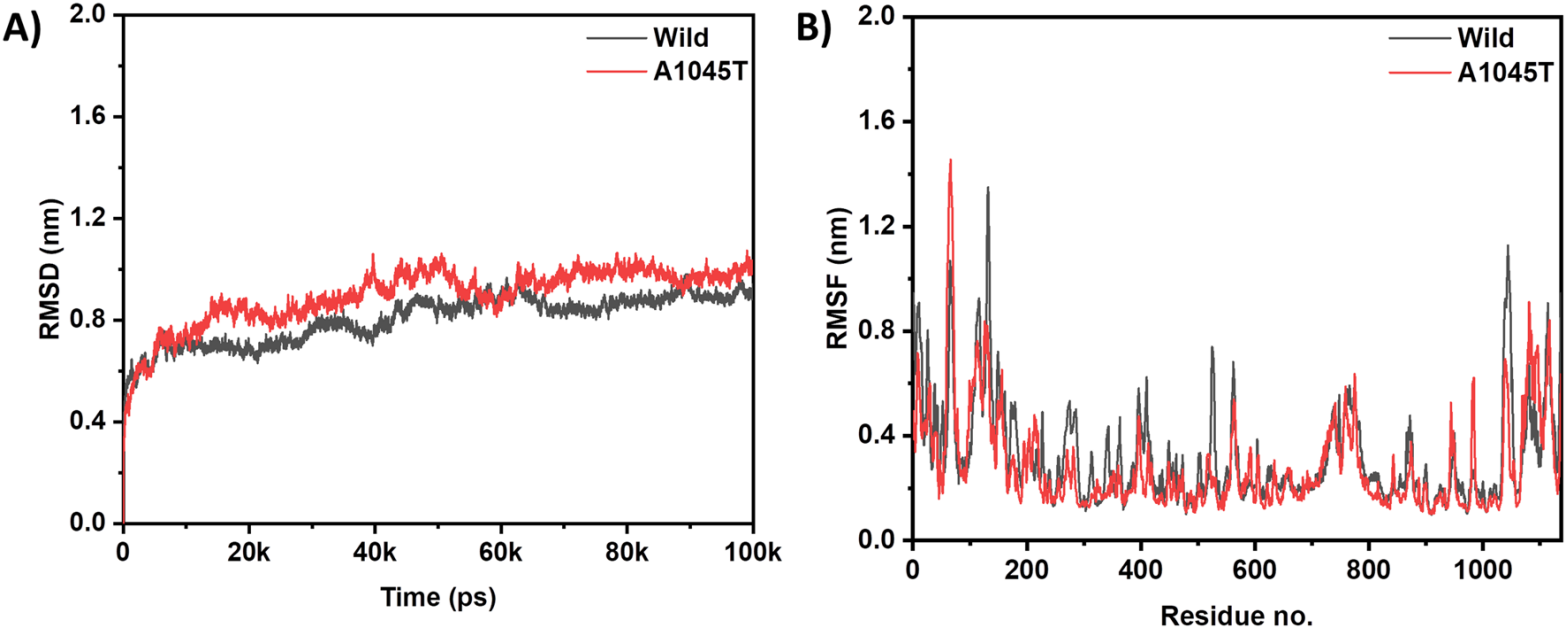
(**A**) RMSD of Wild MSH3 (black) and mutant A1045T MSH3 (red) and (**B**) RMSF of Ca atoms of wild MSH3 (black) and mutant A1045T MSH3 (red).

By measuring the RMSD, we can observe how far atoms have moved from their original positions. The root-mean-squared deviation (RMSD) between the wild type and A1045T mutant is shown in Fig. 3A. It was found that while both exhibit steady and smooth RMSD during the simulation periods, the RMSD of the mutant deviates more from the wild type. This indicates that the mutant is more deviated from its native state. The average RMSD for the wild type and mutant is 0.82 nm and 0.92 nm, respectively. We also determined the RMSF of both wild and mutant Cα-atoms, which monitors their average rate of change during the simulation times. Similar RMSF patterns suggest similar loop position fluctuations (Fig. 3B).

**Figure 4:**
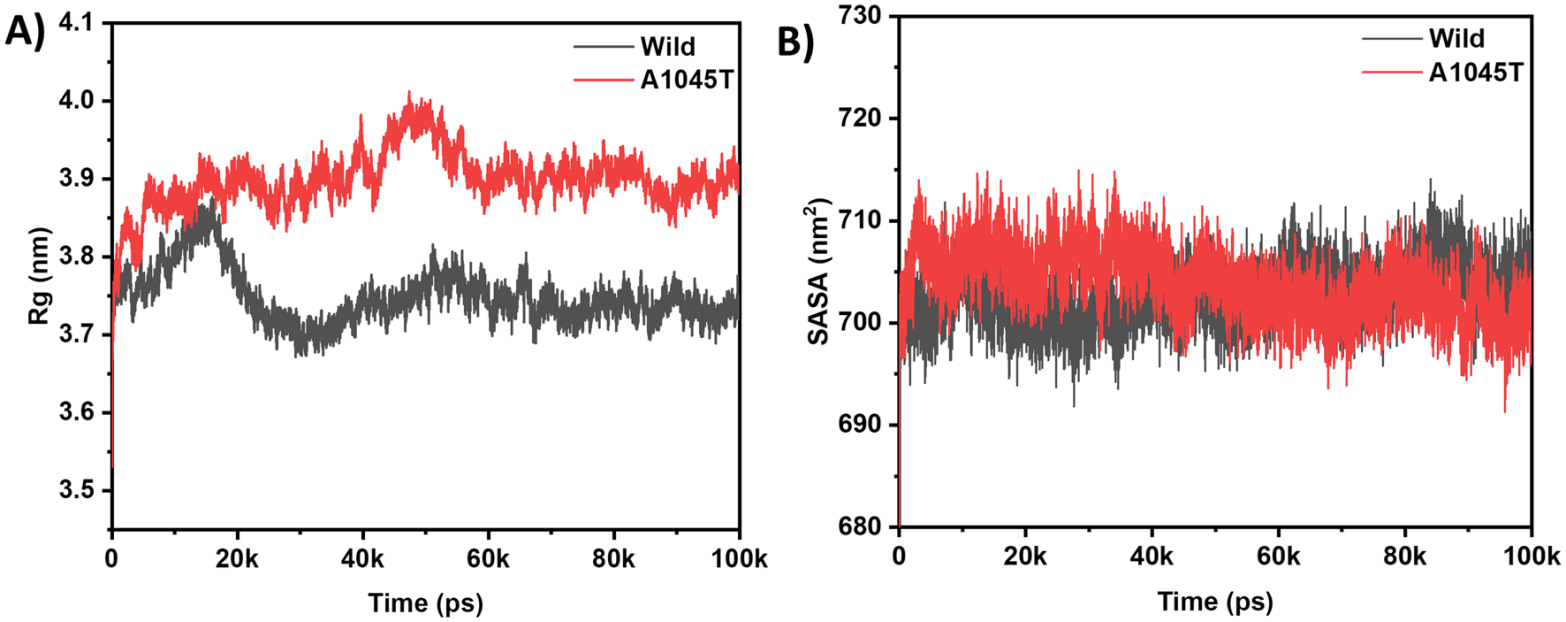
(**A**) Radius of Gyration (Rg), and (**B**) SASA of wild (black) and A1045T mutant (red) form of MSH3 respectively.

The radius of gyration (Rg) measures protein compactness. The Rg value of the mutant is higher and more variable than that of the wild type (Fig. 4A), suggesting that the A1045T mutation has caused a loss of protein compactness. After 40 ns, the wild type’s Rg value is lower and remains steady throughout the simulation.

Similarly, the solvent accessible surface area (SASA) assesses protein stability across various simulation iterations. A lower SASA value indicates greater stability. In this case, the SASA plot for the apo protein closely resembles that of the mutant, with both proteins exhibiting a smooth and stable SASA plot over extended simulation durations (Fig. 4B).

**Figure 5:**
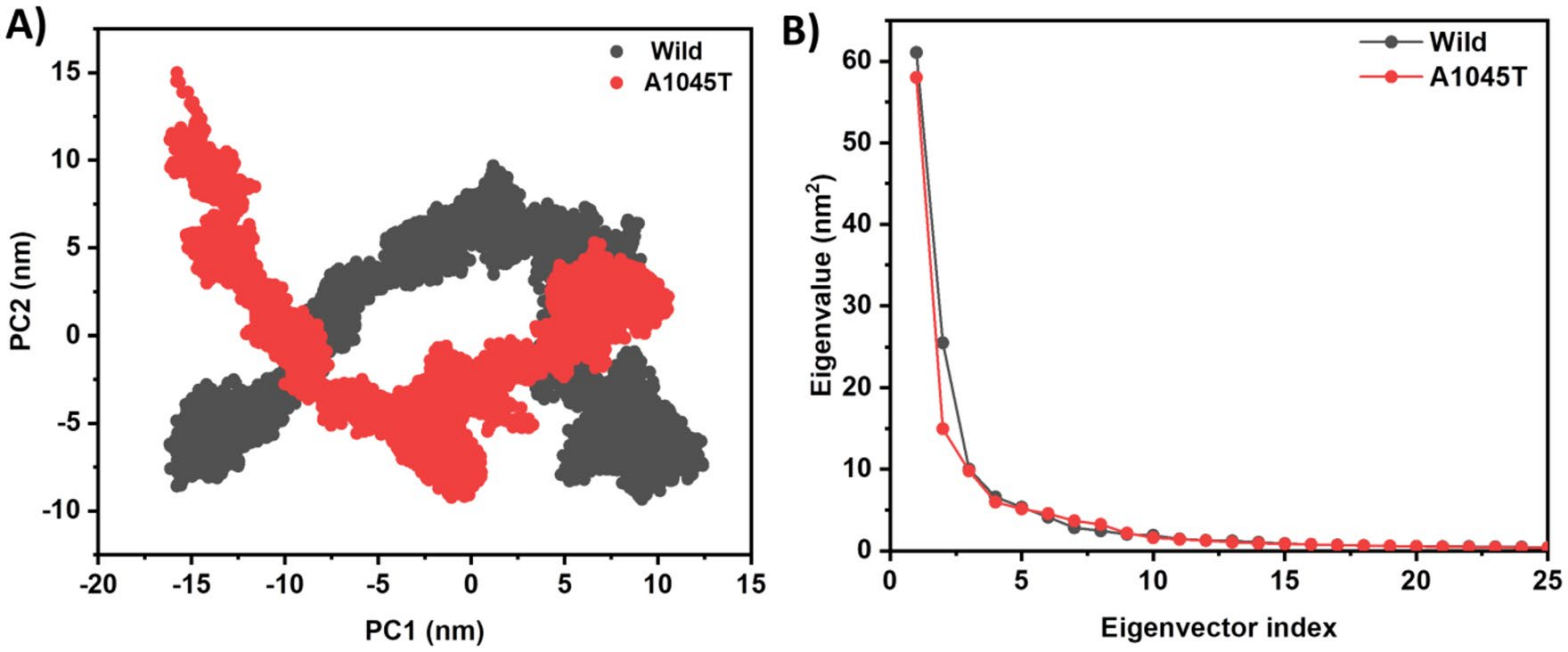
(**A**) 2D projection plots of first two principal eigenvectors and (**B**) Decaying curve of eigenvalue against eigenvector indices coming from covariance matrix, of wild (black) and A1045T mutant (red) respectively.

Figure 5 illustrates the principal component analysis (PCA) results for both apo and mutant MGMT proteins. Cartesian coordinates representing atomic displacements in each trajectory conformation are used to construct a covariance or correlation matrix, reflecting the protein’s available degree of freedom (DOF). Decomposition of the C-matrix into orthogonal collective modes (eigenvectors) enables characterization of each motion component based on its associated eigenvalue (variance), with larger eigenvalues indicating larger spatial scale motions. The two-dimensional projection plot of the first main eigenvectors for both apo and mutant proteins is depicted in Fig. 3A. Throughout the simulation, the mutant protein shares the same subspace with the wild type and exhibits equivalent atomic motion. Eigenvalue versus eigenvector plots for the first 15 modes of the essential subspace, representing 95% of the protein’s variation, are shown in Fig. 5B. The PCA analysis over a 100 ns period indicates that neither protein undergoes significant changes in its atomic coordinates.

## Discussion

The mismatch repair (MMR) pathway is responsible for recognizing and repairing the erroneous insertion, misincorporation and deletion of the bases during replication and recombination. Mutations in DNA repair pathways have been associated with the development of various types of cancer^23–25^. Several studies have addressed whether some genetic variation (SNPs) affects various clinical outcomes in lung cancer patients^23,26^. This case–control study focuses on whether *MSH3 Ala*^*1045*^*Thr* (rs26279) and *MSH6* (rs3136228) genetic polymorphisms play any role in modulating the risk for lung cancer. Furthermore, we also evaluated the impact of these polymorphisms on the outcome of lung cancer patients with platinum-based doublet chemotherapy.

Data from our study suggest a lack of any significant association between *MSH3 Ala*^*1045*^*Thr* polymorphism and the risk of developing lung cancer. As far as our knowledge is concerned, this is the first study to evaluate and analyze the role of *MSH3 rs26279* polymorphism towards the risk of occurrence of lung cancer in North Indians. Our results here are supported by an earlier study which reported no association between *MSH3* (rs26279) polymorphism and risk for lung cancer^15^. Smith and coworkers have also reported no association between rs26279 polymorphism and susceptibility towards breast cancer in the Caucasian population^27^. However, on the contrary, a few studies have found an association between *MSH3 Ala*^*1045*^*Thr* polymorphism with an increased propensity towards colorectal and breast cancer^28,29^. For *MSH6* (rs3136228) polymorphism, our results suggest an increased susceptibility towards lung cancer in subjects harbouring the *GG* genotype (AOR 1.43; *p* = 0.03) when the recessive genetic model was applied. Our findings were further corroborated by results shown in a study conducted by Tulupova and colleagues reporting an increase in susceptibility towards lung cancer in the Czech Republic populace^29^.

Another well-known risk factor for lung cancer is tobacco smoking; therefore, to evaluate the synergistic role of smoking and *MSH3* & *MSH6* polymorphisms towards lung cancer susceptibility, we stratified our data based on smoking status to study the gene-environment interaction. Our data suggest that smoking status does not affect *MSH3 Ala*^*1045*^*Thr* polymorphism and the risk of developing lung cancer. However, a study conducted by Vogelsang and colleagues reported a significant increase in the promoter methylation of MSH3 (91.9%) and further concluded that the factors responsible for this increase are also responsible for the increased risk of oesophagal cancer^30^. They tested 17 MSH3-related CpG sites, and methylation levels at the Cg16401290 site located in the MSH3 promoter region reported a higher methylation level than normal tissues**.** Our results follow the previous study of Xu and coworkers in the Chinese population; they also reported no association between *MSH3 Ala*^*1045*^*Thr* polymorphism and smokers of lung cancer patients^15^. However, Vogelsang and coworkers have reported that smoking and alcohol intake were associated with an increased risk for oesophageal cancer in the South African populace. In their study, authors have compared two populations (Black vs mixed ancestry) and found that Black and mixed ancestry populations have approximately five- and nineteen-times increased risk of oesophageal cancer due to smoking^31^. Carrera and coworkers further corroborated our results for MSH3 Ala1045Thr polymorphism. They also reported that the *MSH3 Ala*^*1045*^*Thr* polymorphism did not show any synergistic correlation for both smokers and non-smokers and was not found to modulate the susceptibility towards lung cancer in the Caucasian population^32^. For *MSH6* polymorphism, our data suggest that in the recessive model for non-smokers, the TT genotype was observed to incur a threefold risk of lung cancer development (*p* = 0.01). However, we could not find any study demonstrating any association among *MSH6* variants, lung cancer susceptibility and smoking status. Further, we have also analyzed if gender is associated with the risk of developing lung cancer. For MSH3 *Ala*^*1045*^*Thr* polymorphism, heterozygous type genotype (GA) in the co-dominant model females has a twofold increased risk of developing lung cancer. However, a previous study conducted by Conde and coworkers differed from our results and concluded that there is no association between rs26279 polymorphism and susceptibility towards breast cancer in Caucasian females^13^. For *MSH6* 557G > T polymorphism, our data show no association of gender with lung cancer susceptibility, but a study conducted by Carrera and coworkers reported an increased risk of lung cancer in males of Spanish Caucasian origin^32^

We also investigated the role of *MSH3 Ala*^*1045*^*Thr* and *MSH6* 557G > T polymorphism based on clinical-pathological features such as stage, tumour size, lymph node invasion and metastasis. Our data suggest no association between clinical pathological features and lung cancer in both polymorphisms. Previous studies conducted by Xu and coworkers have also shown no relationship^15^. However, in one study conducted on the Indian population by Yadav and coworkers, a significant association was found when cancer stages and the size of head and neck squamous cell carcinoma patients were compared. In the recessive model from this study, it was demonstrated that the combined genotype of homozygous wild and heterozygous (GG + GA) has approximately 2.34- and 2.41-fold associations with tumour stage and size, respectively^24^. Lymph node data also showed a slight association in the recessive model of combined genotype.

We have further analyzed the effect on overall survival (OS) due to *MSH3 Ala*^*1045*^*Thr* and *MSH6 557G* > *T* polymorphism based on different histology subtypes of lung cancer, and our results concluded that there is no significant association between OS and *MSH3* rs26279 and *MSH6 557G* > *T* polymorphism. A study conducted by Nogueria and colleagues has concluded that subjects carrying both the wild alleles for *MSH3 Ala*^*1045*^*Thr* polymorphism had terrible OS compared with the patients with the homozygous variant genotype in head and neck squamous cell carcinoma (HNSCC)^33^. However, one study by zanussu and coworkers on prostate cancer patients reported an increase in OS in patients with at least one *T* allele in the Italian population^34^.

In this study, we also evaluated the association of overall survival in lung cancer patients treated with platinum-based doublet chemotherapy. A previous study reported an increase in resistance towards some cytotoxic agents due to overexpression of MSH3 in the promyelocytic leukaemia cell line^6^. Takahashi and colleagues have also suggested that human colon cancer cell lines that have diminished expression of *MSH3* are sensitive to platinum-based treatment^35^. Our results suggested that whether the patient was given Paclitaxel, Irinotecan or Pemetrexed along with carboplatin/cisplatin, the OS remains unaffected by the chemotherapeutic regimen for *MSH3 Ala*^*1045*^*Thr* polymorphisms, whereas *MSH6* polymorphism reported a twofold higher hazard ratio (*p* = 0.03) for docetaxel + carbo/cisplatin combination. However, we could not find any study investigating the role of MSH6 polymorphism in the overall survival of lung cancer patients concerning chemotherapy. A study on colorectal cancer subjects treated with a FOLFOX4 regimen observed an association of the MSH6 *557G* > *T* polymorphism with neutropenia. Thus, it might be highly plausible that this SNP might affect the genotoxic activity in cells which are non-malignant and thus may be modulating the genotoxic effect of FOLFOX4. The MSH6 *557G* > *T* polymorphism is located in the upstream region of the gene and affects the binding capacity of the Sp1 transcription factor, thus leading to low expression of MSH6 and directly resulting in MMR deficiency.

If emphasis is laid on additional MD characteristics like RMSD and Rg, we see that mutant proteins deviate more are less compact.

The population under study in past investigations reported to date was very small, so a significant strength of our present examination is the high number of subjects who were enrolled for this will build the dependability of our investigation. The present investigation primarily focuses on four point’s viz. increased susceptibility, overall survival, response to chemotherapy and clinic-pathological features associated with *MSH3* and *MSH6* polymorphism. Furthermore, we could not find any study investigating the role of *MSH3* and *MSH6* polymorphism in overall survival and platinum-based doublet chemotherapy. In any case, our investigation also has certain limits. To begin with, even though we have picked an enormous population size, subjects under subcategories are low in number, which may be a constraint. Since smoking and its span are crucial for this investigation, differences in smoking propensities and pack years in the population under study were also considered a limitation. Further, the controlled populace recruited in our examination is enlisted from one specific zone, so there may be a chance of choice biasedness.

### Supplementary Information

Supplementary Information.

## Data Availability

Data generated or analyzed during this study are provided in full within the published article.
