# Supplementary material for: Assessing the impact of MSH3 and MSH6 polymorphisms on lung cancer risk in North Indian patients undergoing platinum chemotherapy through molecular dynamics simulation
Source: Sci Rep. 2024 Jul 13;14:16164. doi: 10.1038/s41598-024-67090-x (PMC11246474; doi:10.1038/s41598-024-67090-x)
Supplement: Supplementary file 1 — Supplementary Information. [file 41598_2024_67090_MOESM1_ESM.docx]

# Supplementary Table 1: Relationship of different genotype with the clinical-pathological parameters

| **Genotype**  **rs26279**  **G>A (MSH3)** | **Clinical stage** | | | | **Primary tumor extension** | | | |
| --- | --- | --- | --- | --- | --- | --- | --- | --- |
|  | **III 193 N (%)** | **IV 257 N (%)** | **AOR**  **(95% CI) ^a^** | ***p*^b^** | **T3 85**  **N (%)** | **T4 303 N (%)** | **AOR**  **(95% CI) ^a^** | ***p*^b^** |
| **AA** | 137 (70.9) | 189 (73.5) | 1.00 (Reference) |  | 58 (68.3) | 224 (73.9) | 1.00 (Reference) |  |
| **GA** | 51 (26.5) | 61 (23.8) | 0.86 (0.55-1.36) | 0.53 | 23 (27.0) | 72 (23.8) | 0.77 (0.44-1.35) | 0.36 |
| **GG** | 5 (2.6) | 7 (2.7) | 1.11 (0.29-4.20) | 0.86 | 4 (4.7) | 7 (2.3) | 0.62 (0.16-2.39) | 0.49 |
| **GG+GA** | 56 (13.4) | 68 (26.4) | 1.15 (0.73-1.80) | 0.54 | 27 (31.7) | 79 (26.0) | 1.22 (0.71-2.16) | 0.43 |
|  | **Lymph Node Invasion** | | | | **Metastasis** | | | |
| **rs26279**  **G>A (MSH3)** | **N_x_+N_0_+N_1_**  **102 N (%)** | **N_2_+N_3_+N_4_**  **373 N (%)** | **AOR (95% CI)** | ***p*^b^** | **No**  **210 N (%)** | **Yes**  **265 N (%)** | **AOR (95% CI)** | ***p*^b^** |
| **AA** | 72 (70.5) | 275 (73.7) | 1.00 (Reference) |  | 151 (71.9) | 196 (73.9) | 1.00 (Reference) |  |
| **GA** | 25 (24.6) | 90 (24.2) | 0.94 (0.56-1.58) | 0.83 | 53 (25.3) | 62 (23.4) | 0.88 (0.56-1.38) | 0.60 |
| **GG** | 5 (4.9) | 8 (2.1) | 0.42 (0.12-1.46) | 0.17 | 6 (2.8) | 7 (2.7) | 1.02 (0.29-3.55) | 0.97 |
| **GG+GA** | 30 (29.4) | 98 (26.2) | 1.01 (0.60-1.69) | 0.94 | 59 (28.0) | 69 (26.0) | 1.17 (0.71-1.74) | 0.62 |
|  | **Clinical stage** | | | | **Primary tumor extension** | | | |
| **rs3136228**  **557 G>T (MSH6)** | **III** | **IV** | **AOR (95% CI)** | ***p*^b^** | **T3** | **T4** | **AOR (95% CI)** | ***p*^b^** |
|  | **193 N (%)** | **257 N (%)** |  |  | **85 N (%)** | **303 N (%)** |  |  |
| TT | 166 (86.1) | 221 (85.9) | 1.00 (Reference) |  | 71 (83.5) | 262 (86.4) | 1.00 (Reference) |  |
| GT | 27 (13.9) | 36 (14.1) | 1.06 (0.60-1.86) | 0.82 | 14 (16.5) | 41 (13.6) | 0.76 (0.39-1.48) | 0.42 |
| GG | 0 (0) | 0 (0) | 0 |  | 0 (0) | 0 (0) | 0 |  |
| GT+GG | 27 (13.9) | 36 (14.1) | 1.06 (0.60-1.86) | 0.82 | 14 (16.5) | 41 (13.6) | 0.76 (0.39-1.48) | 0.42 |
|  | **Lymph Node Invasion** | | | | **Metastasis** | | | |
| **rs3136228**  **557 G>T (MSH6)** | **N_x_+N_0_+N_1_** | **N_2_+N_3_+N_4_** | **AOR (95% CI)** | ***p*^b^** | **No** | **Yes** | **AOR (95% CI)** | ***p*^b^** |
|  | **102 N (%)** | **373 N (%)** |  |  | **210 N (%)** | **265 N (%)** |  |  |
| TT | 86 (84.3) | 322 (86.3) | 1.00 (Reference) |  | 179 (85.3) | 229 (86.5) | 1.00 (Reference) |  |
| GT | 16 (15.7) | 51 (13.7) | 0.86 (0.46-1.58) | 0.63 | 31 (14.7) | 36 (13.5) | 0.95 (0.55-1.64) | 0.86 |
| GG | 0 (0) | 0 (0) | 0 |  | 0 (0) | 0 (0) | 0 |  |
| GT+GG | 16 (15.7) | 51 (13.7) | 0.86 (0.46-1.58) | 0.63 | 31 (14.7) | 36 (13.5) | 0.95 (0.55-1.64) | 0.86 |

a)-Adjusted Odds ratios, 95% confidence intervals and their corresponding *p*-values were calculated by logistic regression analysis after adjusting for age, gender. (b)-Two-sided χ2 test for either genotype distribution or allelic frequencies between the cases and controls. The number in bold indicates the significant values in the table

# Supplementary Table 2: Relationship of genotype distribution with clinical-pathological parameters on the basis of response to chemotherapy

| Genotype | Response to chemotherapy | | AOR (95% CI) | *p*^b^ |
| --- | --- | --- | --- | --- |
| MSH3  rs26279  G>A | **CR+PR**  **87, N (%)** | **SD+PD**  **143, N (%)** |  |  |
| AA | 68 (78.2) | 109 (76.3) | 1.00 (Reference) |  |
| GA | 15 (17.3) | 33 (23.0) | 1.43 (0.71-2.88) | 0.30 |
| GG | 4 (4.5) | 1 (0.7) | 0.12 (0.01-1.30) | 0.08 |
| GG+GA | 19 (21.8) | 34 (23.7) | 0.66 (0.32-1.32) | 0.24 |
| Genotype | **Response to chemotherapy** | | **AOR (95% CI)** | ***p*^b^** |
| rs3136228  557 G>T (MSH6) | **CR+PR**  **87, N (%)** | **SD+PD**  **143, N (%)** |  |  |
| TT | 73 (83.9) | 125 (87.5) | 1.00 (Reference) |  |
| GT | 14 (16.1) | 18 (12.5) | 0.76 (0.33-1.55) | 0.78 |
| GG | 0 (0) | 0 (0) | 0 |  |
| GT+GG | 14 (16.1) | 18 (12.5) | 0.76 (0.33-1.55) | 0.78 |

a)-Adjusted Odds ratios, 95% confidence intervals and their corresponding *p*-values were calculated by logistic regression analysis after adjusting for age, gender. (b)-Two-sided χ2 test for either genotype distribution or allelic frequencies between the cases and controls. The number in bold indicates the significant values in the table

# Supplementary Table 3: Association of *MSH3* and MSH6 polymorphism on overall survival in lung cancer cases and on the basis of histology

| **Overall Survival** | | | | | | | | | | | | | | | | | |
| --- | --- | --- | --- | --- | --- | --- | --- | --- | --- | --- | --- | --- | --- | --- | --- | --- | --- |
| **MSH3**  **rs26279**  **G>A** | **Cases**  **N(%),**  **N= 475** | **Dead**  **N(%),**  **N= 356** | **Alive**  **N(%),**  **N= 119** | **Univariate analysis** | | | | | | | **Multivariate analysis** | | | | | | |
|  |  |  |  | **MST (months)** | **Log rank *p*** | | **Unadjusted hr^a^** | | | | **Adjusted hr^b^ (95% CI)** | | | | | ***P*** | |
| **AA** | 349 (73.6) | 259 (72.8) | 90 (75.6) | 8.73 |  | | 1.00 (Reference) | | | |  | | | | |  | |
| **GA** | 112 (23.5) | 89 (25.0) | 23 (19.4) | 6.9 | 0.29 | | 1.13 (0.88-1.45) | | | | 1.13 (0.88-1.46) | | | | | 0.32 | |
| **GG** | 14 (2.9) | 8 (2.2) | 6 (5.0) | 16.7 | 0.14 | | 0.62 (0.32-1.05) | | | | 0.61 (0.27-1.34) | | | | | 0.32 | |
| **GG+GA** | 126 (26.5) | 97 (27.2) | 29 (24.3) | 7.23 | 0.60 | | 1.06 (0.83-1.35) | | | | 1.08 (0.85-1.38) | | | | | 0.50 | |
|  | | | | | | | | | | | | | | | | | |
| **MSH6**  **rs3136228**  **557 G>T** | **Cases**  **N(%),**  **N= 475** | **Dead**  **N(%),**  **N= 356** | **Alive**  **N(%),**  **N= 119** | **Univariate analysis** | | | | | | | **Multivariate analysis** | | | | | | |
|  |  |  |  | **MST (months)** | **Log rank *p*** | | **Unadjusted hr^a^** | | | | **Adjusted hr^b^ (95% CI)** | | | | | ***P*** | |
| TT | 410 (86.3) | 302 (84.8) | 108 (90.7) | 8.66 |  | | 1.00 (Reference) | | | |  | | | | |  | |
| GT | 65 (13.7) | 54 (15.2) | 11 (9.3) | 5.93 | **0.03** | | 1.35 (0.95-1.87) | | | | 1.23 (0.91-1.67) | | | | | 0.17 | |
| GG | 0 (0) | 0 (0) | 0 (0) | 0 | 0 | | 0 | | | | 0 | | | | | 0 | |
| GT+GG | 65 (13.7) | 54 (15.2) | 11 (9.3) | 5.93 | 0.03 | | 1.35 (0.95-1.87) | | | | 1.23 (0.91-1.67) | | | | | 0.17 | |
| **ADCC** | | | | | | | | | | | | | | | | | |
| **MSH3**  **rs26279**  **G>A** | **Cases**  **N(%),**  **N= 191** | **Dead**  **N(%),**  **N= 131** | **Alive**  **N(%),**  **N= 60** | **Univariate analysis** | | | | | | | **Multivariate analysis** | | | | | | |
|  |  |  |  | **MST (months)** | **Log rank *p*** | **Unadjusted hr^a^** | | | **Adjusted hr^b^ (95% CI)** | | | | | ***P*** | | | |
| **AA** | 142 (74.3) | 97 (74.1) | 45 (75.0) | 8.9 |  | | 1.00 (Reference) | | | |  | | | | |  | |
| **GA** | 46 (24.1) | 34 (25.9) | 12 (20) | 7.9 | 0.85 | | 1.03 (0.69-1.53) | | | | 0.96 (0.64-1.45) | | | | | 0.87 | |
| **GG** | 3 (1.6) | 0 (0) | 3 (5.0) | - | 0.06 | | - | | | | 0 | | | | | 0.96 | |
| **GG+GA** | 49 (25.6) | 34 (25.9) | 15 (25.0) | 9.2 | 0.75 | | 0.94 (0.63-1.38) | | | | 0.88 (0.59-1.33) | | | | | 0.56 | |
|  | | | | | | | | | | | | | | | | | |
| **MSH6**  **rs3136228**  **557 G>T** | **Cases**  **N(%),**  **N= 191** | **Dead**  **N(%),**  **N= 131** | **Alive**  **N(%),**  **N= 60** | **Univariate analysis** | | | | | | | **Multivariate analysis** | | | | | | |
|  |  |  |  | **MST (months)** | **Log rank *p*** | | **Unadjusted hr^a^** | | | | **Adjusted hr^b^ (95% CI)** | | | | | ***P*** | |
| TT | 167 (87.5) | 115 (87.7) | 52 (86.7) | 9.13 |  | | 1.00 (Reference) | | | |  | | | | |  | |
| GT | 24 (12.5) | 16 (12.3) | 8 (13.3) | 5.06 | 0.62 | | 1.13 (0.65-1.97) | | | | 1.24 (0.73-2.11) | | | | | 0.41 | |
| GG | 0 (0) | 0 (0) | 0 (0) | 0 | 0 | | 0 | | | | 0 | | | | | 0 | |
| GT+GG | 24 (12.5) | 16 (12.3) | 8 (13.3) | 5.06 | 0.62 | | 1.13 (0.65-1.97) | | | | 1.24 (0.73-2.11) | | | | | 0.41 | |
| **SQCC** | | | | | | | | | | | | | | | | | |
| **MSH3**  **rs26279**  **G>A** | **Cases**  **N(%),**  **N= 202** | **Dead**  **N(%),**  **N= 155** | **Alive**  **N(%),**  **N= 47** | **Univariate analysis** | | | | | | | | **Multivariate analysis** | | | | | |
|  |  |  |  | **MST (months)** | **Log rank *p*** | | | **Unadjusted hr^a^** | | | **Adjusted hr^b^ (95% CI)** | | | | ***P*** | |  |
| AA | 144 (71.3) | 110 (70.9) | 34 (72.4) | 8.6 |  | | 1.00 (Reference) | | | | |  | | | |  | |
| GA | 49 (24.2) | 39 (25.2) | 10 (21.3) | 5.93 | 0.29 | | 1.21 (0.82-1.75) | | | | | 1.38 (0.93-2.03) | | | | 0.10 | |
| GG | 9 (4.45) | 6 (3.9) | 3 (6.3) | 10.56 | 0.57 | | 0.79 (0.37-1.65) | | | | | 0.90 (0.39-2.08) | | | | 0.82 | |
| GG+GA | 58 (28.7) | 45 (29.0) | 13 (27.6) | 5.93 | 0.47 | | 1.13 (0.79-1.62) | | | | | 1.29 (0.90-1.85) | | | | 0.16 | |
| **MSH6**  **rs3136228**  **557 G>T** | **Cases**  **N(%),**  **N= 202** | **Dead**  **N(%),**  **N= 155** | **Alive**  **N(%),**  **N= 47** | **Univariate analysis** | | | | | | | | **Multivariate analysis** | | | | | |
|  |  |  |  | **MST (months)** | **Log rank *p*** | | **Unadjusted hr^a^** | | | | | **Adjusted hr^b^ (95% CI)** | | | | ***P*** | |
| TT | 170 (84.2) | 126 (81.3) | 44 (93.6) | 8 |  | | 1.00 (Reference) | | | | |  | | | |  | |
| GT | 32 (15.8) | 29 (18.7) | 3 (6.4) | 7.65 | 0.11 | | 1.37 (0.87-2.16) | | | | | 0.92 (0.59-1.43) | | | | 0.72 | |
| GG | 0 (0) | 0 (0) | 0 (0) | 0 | 0 | | 0 | | | | | 0 | | | | 0 | |
| GT+GG | 32 (15.8) | 29 (18.7) | 3 (6.4) | 7.65 | 0.11 | | 1.37 (0.87-2.16) | | | | | 0.92 (0.59-1.43) | | | | 0.72 | |
|  | | | | | | | | | | | | | | | | | |
| **SCLC** | | | | | | | | | | | | | | | | | |
| **MSH3**  **rs26279**  **G>A** | **Cases**  **N(%),**  **N= 75** | **Dead**  **N(%),**  **N= 64** | **Alive**  **N(%),**  **N= 11** | **Univariate analysis** | | | | | | | | **Multivariate analysis** | | | | | |
|  |  |  |  | **MST (months)** | **Log rank *p*** | | **Unadjusted hr^a^** | | | **Adjusted hr^b^ (95% CI)** | | | ***P*** | | | | |
| AA | 58 (77.3) | 48 (75.0) | 10 (90.9) | 9.76 |  | | 1.00 (Reference) | | | | |  | | | |  | |
| GA | 16 (21.4) | 15 (23.5) | 1 (9.1) | 6.06 | 0.55 | | 1.19 (0.63-2.24) | | | | | 1.27 (0.67-2.40) | | | | 0.46 | |
| GG | 1 (1.3) | 1 (1.5) | 0 (0) | 6.16 | 0.35 | | 2.47 (0.11-53.2) | | | | | 1.89 (0.21-16.3) | | | | 0.56 | |
| GG+GA | 17 (22.6) | 16 (25.0) | 1 (9.0) | 6.11 | 0.46 | | 1.23 (0.66-2.30) | | | | | 1.29 (0.69-2.41) | | | | 0.41 | |
| **MSH6**  **rs3136228**  **557 G>T** | **Cases**  **N(%),**  **N= 75** | **Dead**  **N(%),**  **N= 64** | **Alive**  **N(%),**  **N= 11** | **Univariate analysis** | | | | | | | | **Multivariate analysis** | | | | | |
|  |  |  |  | **MST (months)** | **Log rank *p*** | | **Unadjusted hr^a^** | | | | | **Adjusted hr^b^ (95% CI)** | | | | ***P*** | |
| TT | 66 (88) | 55 (86) | 11 (100) | 9.58 |  | | 1.00 (Reference) | | | | |  | | | |  | |
| GT | 9 (12) | 9 (14) | 0 (0) | 4.06 | 0.07 | | 1.87 (0.76-4.61) | | | | | 2.09 (0.78-5.59) | | | | 0.14 | |
| GG | 0 (0) | 0 (0) | 0 (0) | 0 | 0 | | 0 | | | | | 0 | | | | 0 | |
| GT+GG | 9 (12) | 9 (14) | 0 (0) | 4.06 | 0.07 | | 1.87 (0.76-4.61) | | | | | 2.09 (0.78-5.59) | | | | 0.14 | |

^a^Unadjusted Hazards ratio for Kaplan meier analysis, ^b^hazards ratio for age, sex, smoking, histology, stage, KPS, ECOG

# Supplementary Table 4: Association of *MSH3* and *MSH6* genotype on survival in lung cancer cases on the basis of chemotherapeutic regimen

| **Docetaxel** | | | | | | | | | |
| --- | --- | --- | --- | --- | --- | --- | --- | --- | --- |
| **Genotype**  **MSH3**  **rs26279**  **G>A** | **CASES**  **n(%), N=36** | **DEAD**  **n(%), N=28** | | **ALIVE**  **n(%), N=8** | **Univariate analysis** | | | **Multivariate analysis** | |
|  |  |  |  |  | **MST (months)** | **Log rank *p*** | **Unadjusted HR^a^** | **Adjusted HR^b^ (95% CI)** | ***P*** |
| AA | 26 (72.2) | 20 (71.5) | | 6 (75) | 8.96 |  | 1.00 (Reference) |  |  |
| GA | 10 (27.8) | 8 (28.5) | | 2 (25) | 8.36 | 0.68 | 1.18 (0.50-2.78) | 0.83 (0.30-2.27) | 0.71 |
| GG | 0 | 0 | | 0 | 0 | 0 | 0 | 0 | 0 |
| GG+GA | 10 (27.8) | 8 (28.5) | | 2 (25) | 8.36 | 0.68 | 1.18 (0.50-2.78) | 0.83 (0.30-2.27) | 0.71 |
| **Genotype**  **MSH6**  **rs3136228**  **557 G>T** | **CASES**  **n(%), N=36** | **DEAD**  **n(%), N=28** | | **ALIVE**  **n(%), N=8** | **Univariate analysis** | | | **Multivariate analysis** | |
|  |  |  |  |  | **MST (months)** | **Log rank *p*** | **Unadjusted HR^a^** | **Adjusted HR^b^ (95% CI)** | ***P*** |
| TT | 30 (83.3) | 22 (78.5) | | 8 (100) | 9.13 |  | 1.00 (Reference) |  |  |
| GT | 6 (16.7) | 6 (21.5) | | 0 | 4.9 | 0.02 | 2.59 (0.73-9.15) | 2.28 (0.59-8.82) | **0.03** |
| GG | 0 | 0 | | 0 | 00 | 0 | 0 | 0 | 0 |
| GT+GG | 6 (16.7) | 6 (21.5) | | 0 | 4.9 | 0.02 | 2.59 (0.73-9.15) | 2.28 (0.59-8.82) | 0.23 |
| **Paclitaxel** | | | | | | | | | |
| **Genotype**  **MSH3**  **rs26279**  **G>A** | **CASES**  **n(%), N=81** | | **DEAD**  **n(%), N=62** | **ALIVE**  **n(%), N=19** | **Univariate analysis** | | | **Multivariate analysis** | |
|  |  |  |  |  | **MST (months)** | **Log rank *p*** | **Unadjusted HR^a^** | **Adjusted HR^b^ (95% CI)** | ***P*** |
| AA | 60 (74.1) | | 46 (74.2) | 14 (73.7) | 5.83 |  | 1.00 (Reference) |  |  |
| GA | 17 (21.0) | | 14 (22.6) | 3 (15.8) | 7.96 | 0.73 | 1.10 (0.59-2.05) | 1.47 (0.74-2.89) | 0.26 |
| GG | 4 (4.9) | | 2 (3.2) | 2 (10.5) | 16.7 | 0.24 | 0.44 (0.16-1.19) | 0.49 (0.11-2.13) | 0.34 |
| GG+GA | 21 (25.9) | | 16 | 5 (26.3) | 8.5 | 0.79 | 0.92 (0.52-1.62) | 1.14 (0.62-2.11) | 0.65 |
| **Genotype**  **MSH6**  **rs3136228**  **557 G>T** | **CASES**  **n(%), N=81** | | **DEAD**  **n(%), N=62** | **ALIVE**  **n(%), N=19** | **Univariate analysis** | | | **Multivariate analysis** | |
|  |  |  |  |  | **MST (months)** | **Log rank *p*** | **Unadjusted HR^a^** | **Adjusted HR^b^ (95% CI)** | ***P*** |
| TT | 69 (85.2) | | 52 (83.9) | 17 (89.4) | 6.9 |  | 1.00 (Reference) |  |  |
| GT | 12 (14.8) | | 10 (16.1) | 2 (10.6) | 5.6 | 0.64 | 1.17 (0.57-2.40) | 0.95 (0.47-1.93) | 0.89 |
| GG | 0 (0) | | 0 (0) | 0 (0) | 0 | 0 | 0 | 0 | 0 |
| GT+GG | 12 (14.8) | | 10 (16.1) | 2 (10.6) | 5.6 | 0.64 | 1.17 (0.57-2.40) | 0.95 (0.47-1.93) | 0.89 |
| **Irinotecan** | | | | | | | | | |
| **Genotype**  **MSH3**  **rs26279**  **G>A** | **CASES**  **n(%), N=41** | **DEAD**  **n(%), N=33** | | **ALIVE**  **n(%), N=8** | **Univariate analysis** | | | **Multivariate analysis** | |
|  |  |  |  |  | **MST (months)** | **Log rank *p*** | **Unadjusted HR^a^** | **Adjusted HR^b^ (95% CI)** | ***P*** |
| AA | 33 (80.5) | 25 (75.8) | | 8 (100) | 10.65 |  | 1.00 (Reference) |  |  |
| GA | 8 (19.5) | 8 (24.2) | | 0 (0) | 5.03 | 0.16 | 1.72 (0.67-4.41) | 1.43 (0.51-4.01) | 0.49 |
| GG | 0 (0) | 0 (0) | | 0 (0) | 0 | 0 | 0 | 0 | 0 |
| GG+GA | 8 (19.5) | 8 (24.2) | | 0 (0) | 5.03 | 0.16 | 1.72 (0.67-4.41) | 1.43 (0.51-4.01) | 0.49 |
| **Genotype**  **MSH6**  **rs3136228**  **557 G>T** | **CASES**  **n(%), N=41** | **DEAD**  **n(%), N=33** | | **ALIVE**  **n(%), N=8** | **Univariate analysis** | | | **Multivariate analysis** | |
|  |  |  |  |  | **MST (months)** | **Log rank *p*** | **Unadjusted HR^a^** | **Adjusted HR^b^ (95% CI)** | ***P*** |
| TT | 36 (87.8) | 28 (84.8) | | 8 (100) | 10.56 |  | 1.00 (Reference) |  |  |
| GT | 5 (12.2) | 5 (15.2) | | 0 | 5 | 0.25 | 1.69 (0.53-5.43) | 1.71 (0.47-6.24) | 0.41 |
| GG | 0 | 0 | | 0 | 0 | 0 | 0 | 0 | 0 |
| GT+GG | 5 (12.2) | 5 (15.2) | | 0 | 5 | 0.25 | 1.69 (0.53-5.43) | 1.71 (0.47-6.24) | 0.41 |
| **Pemetrexed** | | | | | | | | | |
| **Genotype**  **MSH3**  **rs26279**  **G>A** | **CASES**  **n(%), N=105** | **DEAD**  **n(%), N=77** | | **ALIVE**  **n(%), N=28** | **Univariate analysis** | | | **Multivariate analysis** | |
|  |  |  |  |  | **MST (months)** | **Log rank *p*** | **Unadjusted HR^a^** | **Adjusted HR^b^ (95% CI)** | ***P*** |
| AA | 75 (71.5) | 56 (72.7) | | 19 (67.8) | 7.9 |  | 1.00 (Reference) |  |  |
| GA | 27 (25.7) | 20 (25.9) | | 7 (25) | 7.7 | 0.56 | 0.86 (0.52-1.41) | 0.90 (0.52-1.56) | 0.73 |
| GG | 3 (2.8) | 1 (1.4) | | 2 (7.2) | 19.7 | 0.16 | 0.27 (0.09-0.80) | 0 | 0.95 |
| GG+GA | 30 (28.6) | 21 (27.3) | | 9 (32.1) | 11.6 | 0.31 | 0.77 (0.48-1.25) | 0.84 (0.48-1.45) | 0.53 |
| **MSH6**  **rs3136228**  **557 G>T** | **CASES**  **n(%), N=105** | **DEAD**  **n(%), N=77** | | **ALIVE**  **n(%), N=28** | **Univariate analysis** | | | **Multivariate analysis** | |
|  |  |  |  |  | **MST (months)** | **Log rank *p*** | **Unadjusted HR^a^** | **Adjusted HR^b^ (95% CI)** | ***P*** |
| TT | 93 (88.6) | 67 (87) | | 26 (92.9) | 9.13 |  | 1.00 (Reference) |  |  |
| GT | 12 (11.4) | 10 (13) | | 2 (7.1) | 3.9 | 0.15 | 1.61 (0.72-3.60) | 1.33 (0.65-2.68) | 0.42 |
| GG | 0 | 0 | | 0 | 0 | 0 | 0 | 0 | 0 |
| GT+GG | 12 (11.4) | 10 (13) | | 2 (7.1) | 3.9 | 0.15 | 1.61 (0.72-3.60) | 1.33 (0.65-2.68) | 0.42 |
| ^a^Unadjusted Hazards ratio for Kaplan meier analysis, ^b^hazards ratio for age, sex, smoking, histology, stage, ECOG | | | | | | | | | |
